# Improved Results in Paediatric Diabetes Care Using a Quality Registry in an Improvement Collaborative: A Case Study in Sweden

**DOI:** 10.1371/journal.pone.0097875

**Published:** 2014-05-27

**Authors:** Anette Peterson, Lena Hanberger, Karin Åkesson, Mats Bojestig, Boel Andersson Gäre, Ulf Samuelsson

**Affiliations:** 1 Department of Clinical and Experimental Medicine, Division of Pediatrics and Diabetes. Research Center, Linköping University Hospital, Linköping, Sweden; 2 Department of Pediatric, County Hospital Ryhov, Jönköping, Sweden; 3 Jönköping University, School of Health Science, the Jönköping Academy for Improvement of Health and Welfare and Jönköping County Council, Jönköping, Sweden; 4 Division of Nursing Science, Department of Medical and Health Sciences, Linköping University, Linköping, Sweden; University of Florida, United States of America

## Abstract

**Background:**

Several studies show that good metabolic control is important for children and adolescents with type 1 diabetes. In Sweden, there are large differences in mean haemoglobin A1c (HbA1c) in different hospitals and difficulties implementing national guidelines in everyday practice. This study shows how the participation in an improvement collaborative could facilitate improvements in the quality of care by paediatric diabetes teams. The Swedish paediatric diabetes quality registry, SWEDIABKIDS was used as a tool and resource for feedback and outcome measures.

**Methods:**

Twelve teams at paediatric diabetes centres, caring for 30% (2302/7660) of patients in Sweden, participated in an 18-month quality improvement program. Each team defined treatment targets, areas needing improvement, and action plans. The main outcome was the centre patients' mean HbA1c levels, but other clinical variables and change concepts were also studied. Data from the previous six months were compared with the first six months after starting the program, and the long-term follow up after another eleven months.

**Results:**

All centres reduced mean HbA1c during the second and third periods compared with the first. The mean reduction for all was 3·7 mmol/mol (p<0.001), compared with non-participating centres who improved their mean HbA1c with 1·7 mmol/mol during the same period. Many of the participating centres reduced the frequency of severe hypoglycaemia and/or ketoacidosis, and five centres reached their goal of ensuring that all patients had some sort of physical activity at least once weekly. Change concepts were, for example, improved guidelines, appointment planning, informing the patients, improving teamwork and active use of the registry, and health promotion activities.

**Conclusions:**

By involving paediatric diabetes teams in a quality improvement collaborative together with access to a quality register, the quality of paediatric diabetes care can improve, thereby contributing to a reduced risk of late complications for children and adolescents with diabetes.

## Introduction

Several studies, including the Diabetes Control and Complications Trial (DCCT), have shown that improving metabolic control is important to prevent, delay, or slow the progression of long-term complications from diabetes [1]–[3]. In Sweden, children and adolescents with type 1 diabetes are intensively treated following a national management policy according to International Society for Pediatric and Adolescent Diabetes (ISPAD) guidelines [4]. The population is relatively homogenous, and the centres treat all patients in the catchment area without selection. However, there are substantial differences between the patient's mean haemoglobin A1c (HbA1c) reported at the centres. The latest annual registry report (data from 2012) showed a difference of 13·5 mmol/mol, about 1·2% in National Glycohemoglobin Standardization Program/Diabetes Control and Complications Trial (NGPS/DCCT) [5], between the centres with the lowest and highest mean HbA1c. These differences are not explained by clinical variables [6].

Similarly, neither clinical nor treatment variables could explain the persistent differences between centres found by the Hvidoere study group in another large cohort from diabetes centres worldwide [7]–[9]. Possible reasons suggested included a centre's effectiveness in implementing treatment regimens [7], [9] and clearly setting glycaemic targets [10]. Recently, a study within the Swedish paediatric diabetes quality registry, SWEDIABKIDS [5] showed that team members' policies and approaches affect glycaemic control in children and adolescents. One conclusion was that team members need to be aware of their approach and the importance of effective use of the resources within the team [11].

Quality registries enable us to study clinical variables and outcomes of care. SWEDIABKIDS allows each diabetes centre to follow its results and to benchmark with other centres. Data is continuously registered and can also be followed continuously.

Experience within other medical specialties has shown that a systematic quality improvement collaborative in combination with national quality registers can improve clinical results [12], [13].

With this background, members of the steering committee of SWEDIABKIDS invited all paediatric diabetes teams to participate in a quality improvement collaborative aiming to improve and standardize the quality of paediatric diabetes care. SWEDIABKIDS should be used as a tool and resource for outcome measures. It was believed that improvement would be reached by changes in work processes and not by an increased work load.

## Methods

### The Swedish paediatric diabetes quality registry

Outpatient attendance data from all Swedish paediatric diabetes centres (n = 43) are registered in SWEDIABKIDS, a registry established in 2000. The completeness of centres reporting data increased from 32% to 100% from 2000 to 2007. In Sweden, paediatric departments treat all children and adolescents aged 0 to 18 years with diabetes within their catchment areas. Thus, the registry includes data on almost all (around 99%) of the children and adolescents with diabetes in Sweden. Until the end of 2011, the registry includes data from more than 361,000 outpatient visits.

The registry has been web-based since 2008 and is available to all paediatric diabetes centres in Sweden. SWEDIABKIDS is financially supported by the Association of Local Authorities and Regions, SALAR, which represents the interests of Sweden's municipalities, county councils, and regions [14]. SWEDIABKIDS has the status of a national quality registry.

### HbA1c analysis and clinical parameters

All methods used in Sweden are standardized through the External Quality Assurance in Laboratory Medicine in Sweden (EQUALIS). The data on HbA1c obtained from SWEDIABKIDS was derived from capillary blood samples measured with the Bayer/Siemens DCA-2000 analyser or using local laboratory methods. Because the International Federation of Clinical Chemistry (IFCC) reference method has been adopted in Sweden, HbA1c values will be presented as IFCC (mmol/mol) results. For example, 58 mmol/mol (IFFC) corresponds to 7·5% (NGPS/DCCT), whereas 10 mmol/mol is about 0·9%. According to the Swedish guidelines, children with diabetes visit the diabetes centre at least 4 times annually until the age of 18 years. At these visits, HbA1c and other clinical parameters such as insulin dose, weight, height, physical activity and blood pressure are measured. Physical activity is divided in 5 levels: never (level 1), less than one time/week (level 2), one–two times/week (level 3), three–five times/week (level 4) and daily (level 5). Physical activity is defined as activity more the 30 minutes [15].

### The program for improvement of quality of diabetes care

**Figure 1:**
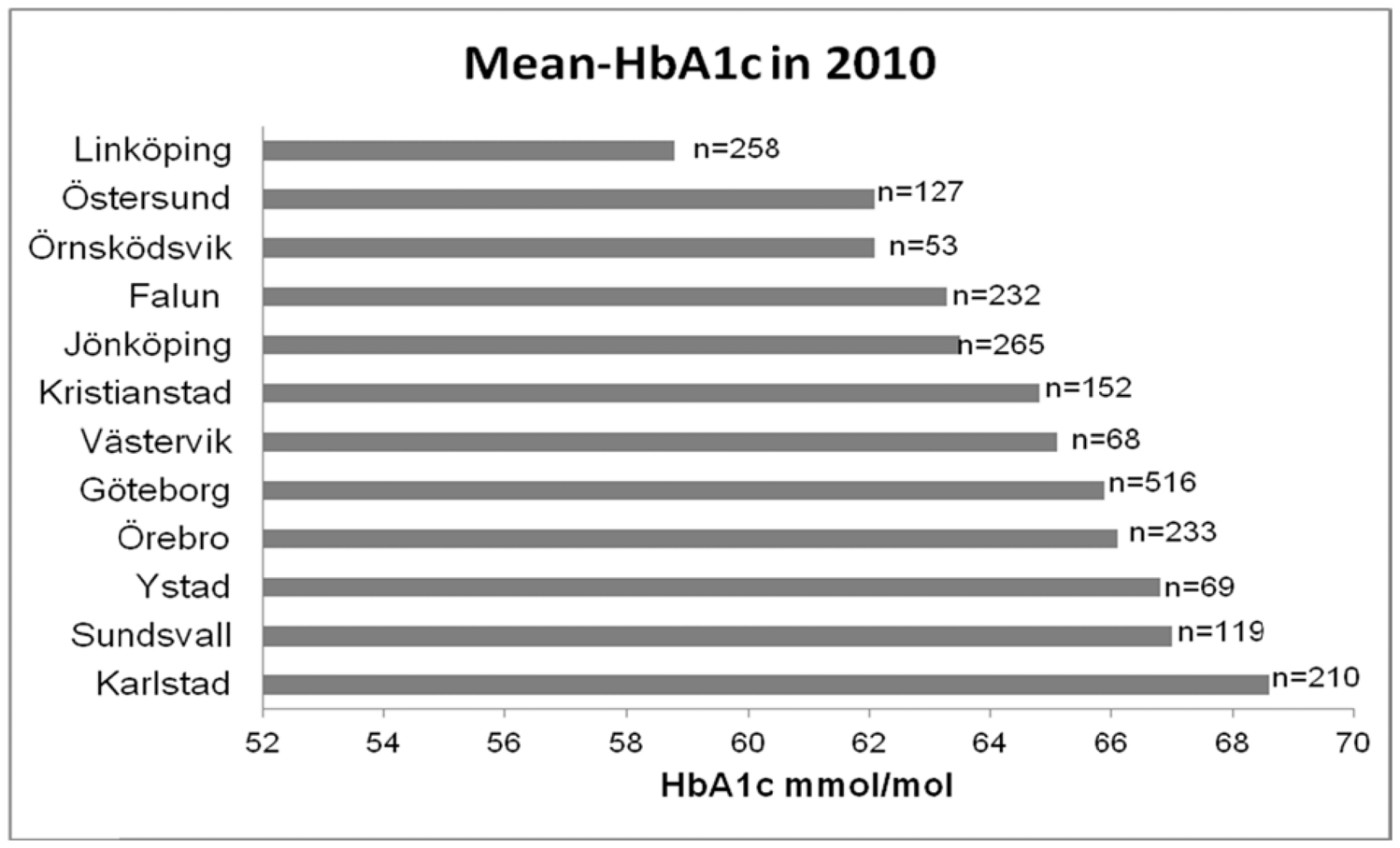
The participating clinics, respective number of patients, and mean HbA1c levels in 2010.

A quality improvement collaborative was conducted in cooperation between SWEDIABKIDS, Qulturum, the Jönköping County Council, and the Jönköping Academy for Improvement of Health and Welfare, Jönköping University. All 43 paediatric diabetes centres in Sweden were invited to participate in the program. Twelve accepted the invitation. In 2010, about 30% (2302/7660) of the patients in Sweden were cared for at these clinics. The number of patients varied between centres from 53 to 516, and the variance in yearly mean HbA1c was between 58·8 and 68·6 mmol/mol (Figure 1).

**Figure 2:**
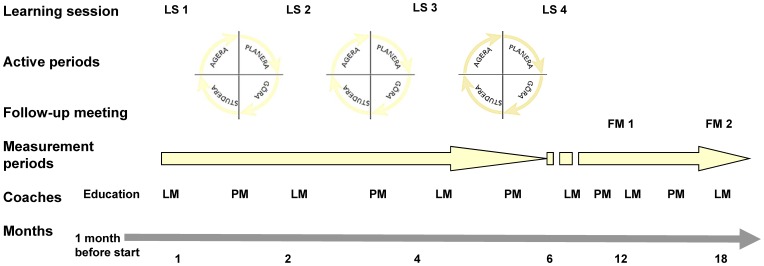
The duration of the collaborative was approximately 18 month with four learning sessions (LS) and two follow up meetings (FM). The team coaches began with a one-day education session followed thereafter by lunch-meetings (LM) at every learning session and phone-meetings (PM) in between.

The improvement collaborative was designed with inspiration from the Breakthrough method [16], [17], included four learning-sessions and two follow-up meetings, and had duration of 18 months (Figure 2). Learning sessions included lectures on improvement methods, teamwork and learning, and sharing experiences between the teams. In the intervals between the learning sessions, the team identified problems and improvement areas at their centres, created action plans, tested changes, and followed up on the results. Most of the improvement work was done at their centre as an integrated part of their own work. The collaborative included learning about and working with systematic improvement methods; for example, the Value Compass, Microsystem analysis, flow charts, fishbone diagrams, and a plan–do–study–act (PDSA) wheel to test different improvement ideas [16], [18]–[20].

In addition to previously used approaches [13], each team also received support from an improvement coach. One member of each team was selected to be the team coach. The coach received extra training and support before the program started in a prophase during the work to effectively support their team at home in the improvement methods, during the action phase, and at the last learning session in a transition phase. The coach should also facilitate the communication with the centres' management to ensure that the work was supported. This coaching model was inspired by the model developed by Godfrey et al [21] for coaching improvement teams in collaborative.

Outcome variables for the project were clinical, processes, and what kinds of change concepts the team used to improve the work. Clinical variables included HbA1c, severe hypoglycaemia, and ketoacidosis. Process measures were documentation of smoking habits and the degree of physical activity. Each participating team was also allowed to define additional targets and outcome variables. Targets defined by specific centres are exemplified in this report as follows: A) to increase the proportion of patients with HbA1c<55 mmol/mol and to decrease the number of patients with HbA1c>70 mmol/mol; and B) to decrease the patients' mean HbA1c and compare it with the mean value of all clinics in Sweden.

To investigate the effect of the quality improvement collaborative, the 6 months prior to the program commencement, November 2010 to April 2011 (period 1), was compared with both the 6-month intensive period (May to October 2011; period 2) and the period thereafter (November 2011 to September 2012; period 3).

At the end of the quality improvement collaborative (October 2012), each team presented a final report. These were analysed with a qualitative content analysis [22] to find themes of change concepts used by the teams to improve their work.

### Statistics

The statistical methods were mostly descriptive. To determine whether changes were significant, Student's T-test was used. A p-value <0.05 was considered significant.

### Ethical consideration

This study does not treat any data for identifiable individual patients; only aggregate data for different health care organizations. The study concerns improvement efforts undertaken by these organizations; not the actions or performance of individuals. Therefore, the study did not require ethical approval in the Swedish system.

## Results

### Project outcome

**Figure 3:**
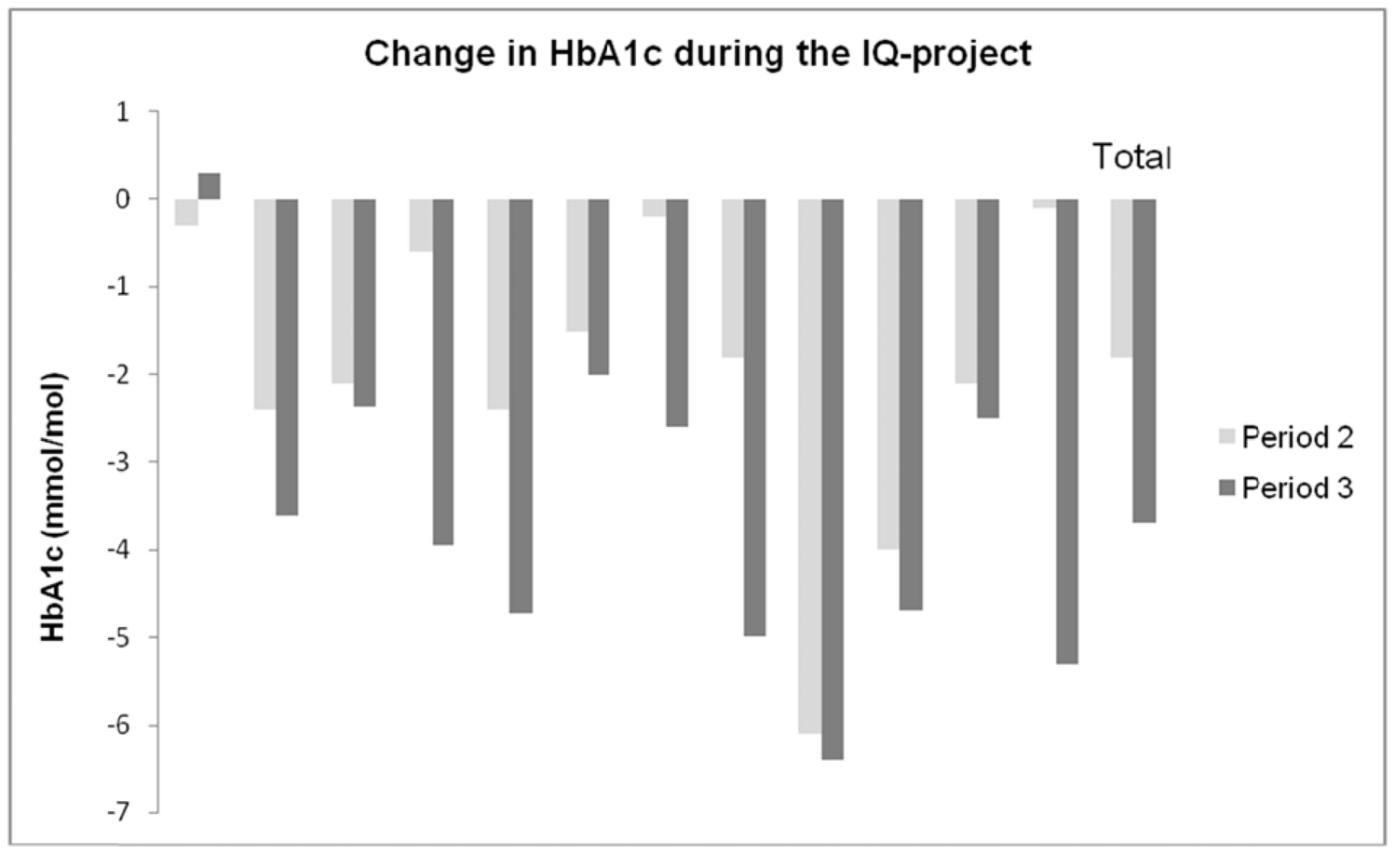
Changes in HbA1c at the different centres between periods 2 and 3 in relation to period 1.

During periods 1, 2 (intensive period), and 3 (follow up period), the centres treated 2,032, 2,004, and 2,119 patients, respectively. At all points of measurement, the mean age of the patients was 13±4.1 years, and 53% were males. As seen in Figure 3, all centres reduced their patients' mean HbA1c in period 2 compared with period 1. The difference was statistically significant for all centres, with a decrease of >1·8 mmol/mol (63·9–62·1 mmol/mol) for the total population (p<0·01). This difference increased further during period 3 to 3·7 mmol/mol (63·9–60·2 mmol/mol) (p<0·001). The difference between period 2 and period 3 was also significant (p<0·01). The 32 clinics who not participated in the project reduced their mean HbAc1 with 0·9 mmol/mol between period 1 and period 2 (64·0–63·1 mmol/mol) and with 1.7 mmol/mol between period 1 and period 3 (64·0–62·3 mmol/mol, p<0·01). Females, in general, had a higher mean HbA1c during all periods compared with males (64.5, 63·1 and 60·6 mmol/mol compared with 62·8, 61·4, and 59·9 mmol/mol, respectively), but the decrease was about the same as for males.

**Figure 4:**
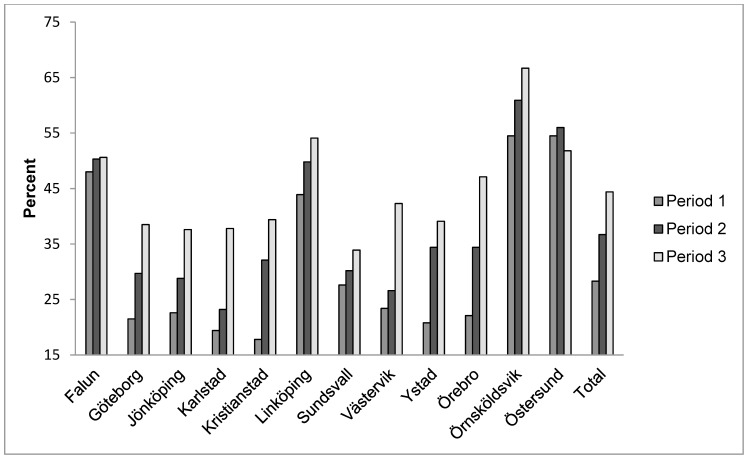
The proportion of patients with a mean HbA1c<57 mmol/mol.

In line with the decreased mean HbA1c during the three periods, the clinics increased the proportion of children with a mean HbA1c<57 mmol/mol from 31·4% in period 1 to 35·5% in period 2 (p<0·05), and 41·1% in period 3 (p<0·01 compared with period 1) (Figure 4). As seen in Figure 4, the centres with a high mean HbA1c at the start of the project/program were the ones that achieved the best improvement in HbA1c. The clinics that not participated increased the proportion of children with mean HbA1c<57 mmol/mol from 28·8% in period 1 to 33·1% in period 2 and 38·4% in period 3 (p<0·001 compared to period 1).

Many of the centres achieved the goal of reducing the frequency of severe hypoglycaemia and/or ketoacidosis (range 0.6%–4.8%). Only two centres increased the frequency (0.9% and 1.4%, respectively). Five of the centres attained the goal of ensuring that all their patients participated in some kind of physical activity of more than 30 minutes duration at least once weekly.

### Process variables

The registration of data on smoking, physical activity, and hypoglycaemia/ketoacidosis increased for most of the participating centres.

### Change concepts

The final reports show six main themes (Table 1) according to the content analysis. Eleven out of 12 teams reported that they developed some activities to improve information, including communication and education to both patients and their families, and to staff members at the centre. All teams improved and updated their local guidelines and procedures, including, for example, routines for complication screening or eye examinations, introducing carbohydrate counting, or insulin pump introduction. Seven teams developed special guidelines for newly-diagnosed diabetes patients, and four developed special activities for patients with high HbA1c, with, for example, more frequent outpatient visits, individual care plans, or direct admission at unacceptably high HbA1c levels. Eight teams improved their reception planning and the same number improved how they used the SWEDIABKIDS registry in clinical work; for example, continuously reviewing statistics. Eight of the teams improved teamwork and six started or improved health promotion activities for their patients (Table 1).

**Table 1:** Theme of the change concepts from the teams' final reports.

Theme of change concepts	Falun	Göteborg	Jönköping	Karlstad	Linköping	Kristianstad	Sundsvall	Västervik	Ystad	Örebro	Örnsköldsvik	Östersund
Information - Communication and education	**x**	**x**		**x**	**x**	**x**	**x**	**x**	**x**	**x**	**x**	**x**
Guidelines/procedures in general	**x**	**x**	**x**	**x**	**x**	**x**	**x**	**x**	**x**	**x**	**x**	**x**
- Special guidelines for new debuted diabetes	**x**			**x**	**x**	**x**	**x**		**x**		**x**	
- Special guidelines for patients with high HbA1c				**x**	**x**		**x**		**x**			
Appointment planning and access	**x**	**x**				**x**	**x**	**x**	**x**		**x**	**x**
Health promotion activities				**x**		**x**		**x**	**x**	**x**		**x**
Improved use of the Registry	**x**		**x**	**x**		**x**		**x**	**x**	**x**		**x**
Improved teamwork			**x**	**x**	**x**	**x**	**x**	**x**	**x**			**x**

## Discussion

The mean HbA1c level was reduced during period 2 when the teams had an intensive period of improvement work. Even more important was that the sustainability of the results was confirmed after another year of long-term follow up. This decrease in mean HbA1c at all participating centres shows the positive influence of the current quality improvement collaborative on the quality of paediatric diabetes care. The centres improved in relation to themselves and to the other centres. The decrease of 1·7 mmol/mol in mean Hba1c at the non-participating clinics could to some degree be secondary to the quality improvement collaborative. This first project was discussed in most of the paediatric diabetes team in Sweden. Many of these teams started on their own to improve their results. This kind of substantial spillover effect on non-enrolled hospitals is known from other studies [23]. A majority of these teams now participate in the second quality improvement collaborative and so far the decrease in HbA1c continuous.

The decrease in mean HbA1c is very encouraging. Many children benefit from this improvement and, if the results are sustainable, have less risk of late complications [1]–[3]. The results emphasize how important it is for health professionals to work continuously and systematically to improve the treatment, structure and processes of care.

Hypoglycaemia can lead to disruptions and practical problems in daily life and have also been found to correlate with lower quality of life [24]. Fear of hypoglycaemia may have a significant negative impact on diabetes management, metabolic control, and subsequent health outcomes [25]. Many of the centres attained or approached their goals of reduced frequencies of severe hypoglycaemia and/or ketoacidosis, and of ensuring that all their patients engaged in some sort of physical activity at least once weekly. There are clinicians who fear that decreasing HbA1c values increase the risk of severe hypoglycaemia. This project shows the opposite pattern. The benefit of physical activity for children and adolescents with diabetes includes better blood glucose control and enhanced insulin sensitivity [26].

Some centres chose their own specific outcome variables, which were easily collected in SWEDIABKIDS; e.g. collecting data on the proportion of patients with low and high HbA1c and comparing the centre's mean HbA1c with the mean HbA1c in Sweden. The result of this study confirm that uniform, simple, and reliable measurements together with a systematic quality improvement stimulate team members and facilitate compliance with the activity plan or changes that the team has set up [27].

The final reports from the teams showed a high level of activity by the team members. Time at the seminars reserved for discussions within the teams provided opportunities to reach agreements on treatment issues, patient education, and treatment targets. In this way, the collaborative combined professional and improvement knowledge to improve the care for the patients, an approach which has been argued by Batalden and Stoltz [20].

Compared with previous collaborative programs [13], the concept of having a coach in each team was developed, a model inspired from the coaching model developed by Godfrey et al [21]. The evaluation of the coaching will be presented in a separate publication.

Some teams focused on the message to patients and reached agreement on the information to be conveyed to families. These factors have been found to be important for successful treatment and adherence to care plans [10], [11].

Furthermore, the collaborative contributed to improving the completeness of data reported to the registry. It also contributed to validating the data in the registry by means of the discussions at the seminars. This led to better conditions for auditing and developing paediatric diabetes care.

In summary, team members can support to decrease patients' mean HbA1c values for the group they serve. We have in this study shown that the access to a quality register to report data, receiving continuous feedback, and being able to compare the centre's own results over time transparently with other centres are important for successful improvement. Together with systematic work in a quality collaborative with support from a coach improvements can be achieved. Health professionals need to continuously work to improve the quality of paediatric diabetes care to reduce the risk of acute and late complications. Involving paediatric diabetes teams in a quality improvement collaborative can help the teams to improve important clinical results.
